# Connecting Pharmacists and Other Health Care Providers (HCPs) towards Drug Therapy Optimization: A Pharmaceutical Care Approach

**DOI:** 10.1155/2023/3336736

**Published:** 2023-01-14

**Authors:** Mir Javid Iqbal, Geer Mohammad Ishaq, Abdullah A. Assiri

**Affiliations:** ^1^Department of Pharmaceutical Sciences, College of Pharmacy, Northeastern University, Boston, USA; ^2^Department of Pharmaceutical Sciences, University of Kashmir, Srinagar, India; ^3^Department of Clinical Pharmacy, College of Pharmacy, King Khalid University, Abha 62529, Saudi Arabia

## Abstract

**Background:**

Pharmaceutical care services offered by pharmacists rationalize drug therapy, improve patient quality of life, and save patients' lives. This study was designed to optimize patient drug therapy through pharmaceutical care services offered by a pharmacist in consultation with other health care providers (HCPs) at a tertiary care hospital.

**Methods:**

This descriptive study was conducted to assess the role and effectiveness of pharmacists in optimizing drug therapy outcomes. The study was carried out at an internal and pulmonary medicine unit of a tertiary care hospital in Srinagar, Jammu and Kashmir, India, with a total of 50 health care providers (HCPs) (24 doctors, 16 nurses, and 10 pharmacists). A total of 182 patients (males and females) of all age groups were recruited into the study over a period of nine months. Patient-specific pharmaceutical care plans initiated by the pharmacist based on drug therapy-related needs and problems were used to address and optimize drug therapy outcomes in consultation with other HCPs.

**Results:**

A total of 388 drug-related problems (DRPs) with an average of 2.29 DRPs per patient were identified, for which 258 pharmaceutical care plans as interventions were proposed, out of which 233 (90.31%) were accepted and implemented. Preassessment and postassessment by HCPs on services rendered by the pharmacist showed a positive change in attitude among HCPs with respect to their endorsement and acceptance of the pharmacist's services in providing direct patient care.

**Conclusions:**

Pharmaceutical care services offered by pharmacists helped in optimizing drug therapy and patient care.

## 1. Introduction

Clinical pharmacists are specially trained practitioners who provide direct patient care by offering services like pharmaceutical care [1]. Pharmaceutical care is the direct, responsible provision of medication-related care wherein the pharmacist works in collaboration with other HCPs and patients to optimize drug therapy outcomes [2, 3]. The concept of care, therefore, emphasizes establishing relationships with other HCPs and patients to achieve desired drug therapy outcomes. However, this concept of care can only be achieved if pharmacists and other HCPs agree on each other's roles because the perceptions of HCPs regarding the pharmacist's role can reduce the level of their cooperation [4, 5]. As the number of pharmacists rendering clinical services per occupied bed increases, mortality, drug costs, total costs of care, and length of stay decrease [5, 6]. The clinical and economic outcomes from a group of patients who received pharmaceutical care showed that, upon first assessment by the pharmaceutical care practitioner, more than fifty percent of the patients had one or more drug therapy problems identified and resolved. Moreover, the healthcare savings realized from pharmaceutical care were found to be huge [7, 8]. At the time of this study, there were no data available in published literature barring one study [9] on pharmacist-led services or interventions offered at any public health facilities in the region of Jammu and Kashmir. This concept has yet to take off in any of the healthcare facilities here, and pharmacists are still viewed as mere drug dispensers by patients and other HCPs alike. Given this dismal scenario, a beginning was made by this study to highlight the role of pharmacists working with other HCPs in providing direct patient care at a tertiary care hospital.

## 2. Methods

### 2.1. Study Design

This descriptive study was carried out for a period of nine months at an in-patient department of a tertiary care hospital in Srinagar, Jammu and Kashmir state, India. The study was conducted to assess the role and effectiveness of pharmacists in optimizing drug therapy outcomes.

### 2.2. Study Population

Study population included HCPs and patients of the super specialty respiratory medicine unit of the study hospital.

### 2.3. Inclusion Criteria

All patients, including both males and females, belonging to all age groups, admitted to the internal and pulmonary medicine units, and having consented to participate, were included in the study.

### 2.4. Methods

At the outset, a preassessment of the HCPs in the study unit was conducted to determine their attitudes and perceptions toward the role of pharmacists in providing direct patient care. The assessment was conducted using a self-administered, prevalidated questionnaire that was internally developed and validated using Delphi and the field pretest method [10, 11].

Following this, a patient-specific continuous assessment by the pharmacist was carried out on a daily basis. All medical and medication information was collected by the pharmacist through patient/caregiver interviews, patient case notes, medication charts, and laboratory data. Patient-specific data were assessed, and all drug therapy-related needs and problems were identified and classified using PCNE criteria. Those drug-related needs and problems identified through the assessment findings were presented, discussed, and validated by attending HCPs. To manage patients' medical conditions, pharmaceutical care action plans were developed by the pharmacist in consultation with the attending physician, which included interventions to address patients' drug-related needs, resolve drug therapy problems, and achieve the goals of therapy. The pharmacotherapy interventions included initiating new drug therapy, discontinuing existing drug therapy, changing the product and/or dosage regimen, and patient education. All interventions were adopted, implemented, followed, and documented in accordance with the revised criteria provided by the Pharmaceutical Care Network Europe and the American Society of Health-System Pharmacists [12, 13].

### 2.5. Ethical Standards

Necessary ethical clearance was obtained from the Institutional Ethics Committee vide reference number SIMS 131/IEC-SKIMS/11-4579. All volunteers were educated about the purpose of the study.

### 2.6. Statistical Analysis

Data were analyzed using SPSS version 24. Descriptive analysis was done using means. A Student's *t*-test was used to evaluate prepharmacist and postpharmacist-led interventions in attitude and perception scores.

## 3. Results

A total of 182 patients and 50 HCPs from the study unit were included in the study. Most patients (53.8%) were aged between 60 and 70 years, and most HCPs were doctors (Table 1). The prepharmaceutical care services assessment revealed that there is a lack of knowledge and negative attitudes and perceptions among HCPs about pharmacy and a pharmacist's role in improving patient health, reducing medication errors, rendering patient counseling, and providing drug information. Only 12% of HCPs agreed that clinical pharmacy services will improve patients' health, and only 10% agreed to accept the involvement of pharmacists in the drug and disease management of patients.

**Figure 1:**
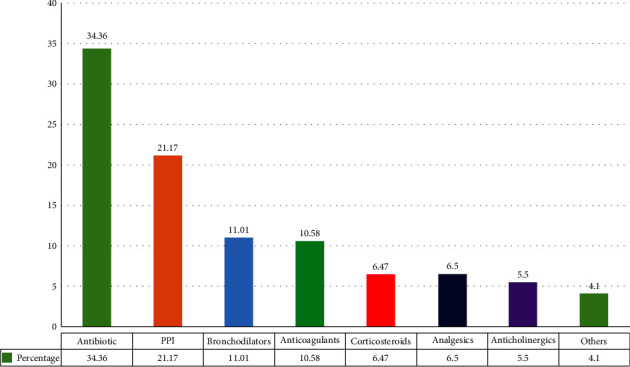
Most frequently used drugs during the study period (*N* = 1260).

Services like pharmaceutical care, patient counseling, and drug information were not rendered prior to this study (Table 2). The patient-specific assessment revealed that a total of 1260 medications were prescribed for 182 patients, with an average of 6.92 drugs per patient. Antibiotics were identified as the most prescribed drugs at 34.36%, followed by proton pump inhibitors (PPIs) at 21.17% (Figure 1). A total of 388 DRPs in 182 patients were identified, with an average of 2.29 DRPs per patient. Patient-specific health-related problems were mainly associated with the drug use process (Table 3). All drug-related problems encountered and all drug-related needs identified during the assessment were addressed, and corresponding action plans were imparted in consultation with the attending physicians and nurses. For the 388 DRPs, 258 interventions were proposed, out of which 230 interventions were accepted and imparted.

A total of 65 interventions were imparted at the patient level, 75 at the prescriber level, and 93 at the drug level (Table 4). Among the drug-related needs of study patients, gastric discomfort, hypokalemia, tachycardia constipation, and oral fungal infections were the most common conditions requiring therapeutic interventions (Table 5). Postassessment by HCPs of services rendered by the pharmacist in providing direct patient care revealed the services to be beneficial and effective in managing patients' drug therapy. All HCPs agreed that, as a healthcare team, they do accept the involvement of pharmacists in the drug and disease management of patients.

## 4. Discussion

Trained pharmacists are best suited to assist HCPs and patients in the prevention of drug-use problems and to achieve optimal and desired outcomes from drug intake [14]. In developing countries, irrational prescribing, dispensing, and administration practices have long been reported [15–18]. Our study provided some insight into the use of medicines by HCPs and patients, including factors affecting drug therapy outcomes. Several published studies have reported the existence of a communication gap between pharmacists and doctors [19, 20] and that the acceptance of pharmacists providing direct patient services is dependent on physician perceptions of pharmacist competence [18, 21, 22].

Pharmaceutical care services rendered through our study were designed to bridge the existing gaps among HCPs and patients so that effective communication is utilized, managed and coordinated care is delivered, and drug therapy outcomes are improved. Drug administration-related errors, including patient noncompliance, accounted for the largest proportion (33.76%) of observed DRPs. Factors that could be responsible for such errors include communication deficits among HCPs and patients, a lack of knowledge, inadequate manpower, and a lack of time to counsel patients. It has been reported that pharmaceutical care appears to increase patient awareness about medication side effects [23]. The present study proposed 258 pharmacist-led interventions, out of which 233 interventions were accepted and implemented. Physicians who collaborate with pharmaceutical care practitioners have validated the work of the practitioners, and patients are also recognizing the benefits of pharmaceutical care [24, 25]. In these ways, pharmaceutical care intervention has stimulated greater involvement with the health care system, thus increasing utilization and improving the quality of patient care.

This study marked the beginning of pharmaceutical care services being offered by a trained pharmacy practitioner. As evident from the results, 80% of HCPs agreed that all necessary and important services under pharmaceutical care were rendered at the patient and prescriber levels. Moreover, 100% of the participants agreed that, as a healthcare team, they do accept the involvement of pharmacists in drug and disease management. Given the changing role of pharmacists and pharmacy automation, physicians need to be proactive and find new ways of collaborating with pharmacists to enhance patient safety and care. Working directly with pharmacists, particularly in medication errors, is one way to do that. When physicians, pharmacists, and patients share responsibility for care, powerfully good results are obtained [26–29].

Pharmaceutical care is a much-needed service, especially in our setting, and therefore it is high time to recognize the need for and importance of implementing such services on the ground. The present scenario in Jammu and Kashmir state, characterized by heavy doctor workloads, deficits in providing drug information, education, and counseling services to patients, and increased use of drugs, is ripe for trained pharmacists to play greater roles, and one such avenue is by offering pharmaceutical care services at both clinical and community levels.

### 4.1. Strengths and Limitations

Our study was the first of its kind in the entire state of Jammu and Kashmir and tried to introduce the concept of pharmaceutical care in a hospital for the first time on a pilot scale. However, our study had several limitations, and therefore, further research is warranted to fill the identified gaps. The study was conducted for a brief period of nine months and was restricted to specific patients of one hospital; therefore, the results and interpretations are limited to the study hospital only. The inclusion of these services in other hospitals would have increased the number of both interventions and outcomes. Disease outcome measures and patient follow-up after discharge were not included in the study because of time constraints. Additionally, the costs, quantitative preventability, and predictability of DRPs were not measured.

## 5. Conclusion

Pharmacist-led pharmaceutical care services were rendered for the first time at the study hospital. As evident from the results, pharmacist-led interventions helped in the optimization of drug therapy through coordinated care with other HCPs. With better professional services and skills, pharmacists can become more acceptable to other HCPs as an integral part of the healthcare team. Healthcare facilities in the developing world need to keep pace with fast-changing trends by establishing departments of clinical pharmacy and involving pharmacists in medical ward rounds so that drug-related problems can be minimized, patients' drug-related needs can be addressed in a better manner, patients can be counseled on their prescribed therapies, and the clinical outcomes of treatments can thereby be maximized.

## Figures and Tables

**Table 1:** Demographic details of the study patients and HCPs.

Age in years	Male	Female	Total
	*N* (%)	*N* (%)	*N* (%)
*Study patients*			
13–19	02 (1.0)	1 (0.5)	03 (1.6)
20–59	48 (26.3)	33 (18.6)	81 (44.5)
60–70	71 (39.0)	27 (14.8)	98 (53.8)
Total	**121 (66.4)**	**61 (33.5)**	**182 (100)**

*Health care providers (HCPs)*			
Doctors	22 (44)	2 (4)	24 (48)
Nurses	3 (6)	13 (26)	16 (32)
Pharmacists	7 (14)	3 (6)	10 (20)
Total	**32 (64)**	**18 (36)**	**50 (100)**

Bold referring to the total.

**Table 2:** HCPs assessment of services during prepharmacistand postpharmacist-led interventions (*N* = 50).

Statements	Percentage agreement		
	Preintervention, *N* (%)	Postintervention, *N* (%)	*P* value, *N* (%)
Do clinical pharmacy services help in improving patients' health?	12 (23.5)	50 (100)	<0.001
Pharmaceutical care services address patients' drug needs and problems	20 (39.2)	43 (84.3)	<0.001
Pharmacists help to optimize patients' drug therapy with other HCPs	14 (27.7)	45 (88.2)	<0.001
Pharmacists should give reliable drug information to HCPs	5 (9.8)	45 (88.2)	<0.001
Pharmacists should counsel patients on the appropriate use of drugs	22 (43.1)	49 (96)	<0.001
Pharmacists should give cost-effective alternatives whenever required	22 (43.1)	38 (74.5)	<0.001
A pharmacist can help in minimizing medication errors in the ward	15 (51)	49 (96)	<0.001
Pharmacists can help in improving the quality of patient care in the ward	20 (39.2)	50 (100)	<0.001
A pharmacist can help as facilitator among HCPs and patients	12 (23.5)	50 (100)	<0.001
HCPs will accept the involvement of pharmacists in the drug and disease management of patients	10 (19.6)	50 (100)	<0.001

**Table 3:** Cause of DRPs as per PCNE classification.

Types	Cause of DRP	*N*	%
Drug selection	Inappropriate drug (including contra-indicated)	2	0.51
	No indication for drug	0	0.0
	Inappropriate combination of drugs, or drugs and food	7	1.80
	Inappropriate duplication of therapeutic group	8	2.06
	Indication for drug treatment not noticed	0	0
	Too many drugs prescribed for indication	3	0.07
	More cost-effective drug available	11	2.83
	Synergistic/preventive drug required and not given	24	6.18
	New indication for drug treatment presented	0	0
	Total	**55**	**14.01**

Drug form	Inappropriate drug form	3	0.07

Dose selection	Drug dose too low	1	0.25
	Drug dose too high	3	0.07
	Dosage regimen not frequent enough	8	2.06
	Dosage regimen too frequent	12	3.09
	No therapeutic drug monitoring	4	1.03
	Pharmacokinetic problem requiring dose adjustment	0	0
	Improvement of disease state requiring dose adjustment	13	3.35
	Total	**44**	**11.34**

Treatment duration	Duration of treatment too short	0	0
	Duration of treatment too long	11	2.83
	Total	**11**	**2.83**

Drug use process	Inappropriate timing of administration or dosing intervals	26	6.70
	Drug underused/underadministered	12	3.09
	Drug overused/overadministered	15	3.86
	Drug not taken/administered at all	24	6.18
	Wrong drug taken/administered	11	2.83
	Drug abused (unregulated overuse)	2	0.51
	Patient unable to use drug/form as directed	41	10.56
	Total	**131**	**33.76**

Logistics	Prescribed drug not available	7	1.80
	Prescribing error (necessary information missing)	7	1.80
	Dispensing error (wrong drug or dose dispensed)	28	7.21
	Total	**42**	**10.82**

Patient	Patient forgets to use/take drug/noncompliant	51	13.14
	Patient uses unnecessary drug	22	5.70
	Patient takes food that interacts	2	0.51
	Patient stored drug inappropriately	14	3.60
	Patient dissatisfied with therapy despite optimal clinical and economic treatment outcomes	3	0.80
	Total	**92**	**23.71**

Other	Adverse drug event	10	2.57
	Drug treatment more costly than necessary	3	0.77
	Total	**13**	**3.35**

Grand total		**388**	**100%**

**Table 4:** Pharmacist-led interventions at various levels during the study period.

Level of intervention	Type of pharmacist led intervention	*N*	% age
(1) At the prescriber level	Prescriber informed only	03	1.16
	Prescriber asked for information	04	1.55
	Intervention approved by prescriber	48	18.9
	Intervention not approved by prescriber	12	4.65
	Intervention proposed, outcome unknown	08	3.10
	Total	**75**	**29.1**

(2) At the patient level	Written information provided	14	5.42
	Patient referred to the prescriber	21	8.13
	Spoken to family member/caregiver	30	14.62
	Total	**65**	**25.2**

(3) At the drug level	Drug changed	08	3.10
	Dosage changed	19	**7.**36
	Formulation changed	02	0.77
	Instructions for use changed	41	15.89
	Drug stopped	09	3.48
	New drug started	14	5.42
	Total	**93**	**36.0**

(4) Other intervention	Other interventions at the patient level	15	5.81
	Side effects reported to authorities	10	3.87
	Total	**25**	**09.7**

Total interventions made at various levels		**258**	**100**

Bold referring to the total.

**Table 5:** Most common DRPs and their management during the study period.

DRPs	No. of events, *N*(%)	Identified cause	Management
Gastric discomfort	108 (46.95%)	Oral antibiotics NSAIDs	Gastric protection/counseling
Hypokalemia	51 (22.17%)	Diuretics	Supplements/diet management
Tachycardia	47 (20.43%	Beta-agonists	Selective agonists
Constipation	46 (22.17%)	Anticholinergics	Dietary counseling
Fungal infection/dryness of mouth	44 (19.13%)	Steroid use/nebulization	Mouth gargles and oral hygiene counseling

## Data Availability

Data are available upon request to the corresponding author.
